# Effect of social support on anxiety of medical staff one year after COVID-19 outbreak: a moderated mediating model

**DOI:** 10.1038/s41598-022-25126-0

**Published:** 2022-12-14

**Authors:** Bin Wang, Xiao Zhong, Haojie Fu, Ruilin Hu, Mengting He, Guanzi Zhang

**Affiliations:** 1 Psychosocial Service and Crisis Intervention Research Centre, Southwest University of Science and Technology, East Building 7-409, No. 59 of Qinglong Street, Fucheng District, Mianyang, 621010 Sichuan Province China; 2 Department of Psychology, Beijing Sport University, Beijing, China; 3 Shanghai Research Institute for Intelligent Autonomous Systems, Tongji University, Shanghai, China; 4 School of Foreign Languages and Cultures, Southwest University of Science and Technology, East Building 7-409, No. 59 of Qinglong Street, Fucheng District, Mianyang, 621010 Sichuan Province China

**Keywords:** Health care, Medical research

## Abstract

One year after the outbreak of COVID-19, medical staff are facing high anxiety due to multiple work stresses. Social support has become a protective factor for healthcare workers' anxiety symptoms, but the exact mechanism of action is not yet known. Based on the buffering model, this study aims to explore the impact and mechanisms of action of healthcare workers' perception of social support (PSS) on anxiety symptoms in the context of the epidemic and to further explore how the emotional characteristics of risk perception (ECRP) and resilience play their influence. To this end, this study measured 839 healthcare workers using an online questionnaire from 4 February to 1 March 2021. The results found that PSS among healthcare workers negatively predicted anxiety symptoms. ECRP partially mediated the relationship between PSS and anxiety symptoms, and resilience moderated the first half of the pathway in the model of PSS through ECRP on anxiety symptoms. The emotional characteristics of risk perception of COVID-19 in individuals with high resilience decrease significantly with the increase of PSS, while this change is not significant in individuals with low resilience.

## Introduction

In early 2020, a large outbreak of COVID-19 spread worldwide, which impact global mental health^1^. While medical workers, as the hub of epidemic prevention and control, are faced with multiple stressors and are prone to intense anxiety symptoms^2^. A recent global survey on physician mental health showed that 32.8%, 30.8%, 25.9%, and 24.0% of participants screened positive for depression, anxiety, stress, and post-traumatic stress disorder^3^. Most of the previous studies have explored the factors that contribute to the deterioration of the mental health of health workers during pandemics, such as Wang et al. found that physical symptoms of COVID-19 infection can lead to poor mental health outcomes in individuals through the need for health information and the perception of pandemic impact^4^. Researchers have paid less attention to the question of what factors currently help protect their mental health. The exploration of protective factors for the mental health of medical workers is also an important research orientation for psychological interventions.

Good interpersonal support is an important protective factor for mental health. Perceived social support (PSS) is defined as an individual's subjective perception and evaluation that he or she is supported, understood and respected by the outside world^5,6^. According to the social support buffer model^7^, social support provides individuals with the resources to cope with traumatic events, which contributes to the enhancement of individuals' self-worth, thus mitigating the emotional impact of traumatic events on individuals. During the epidemic, there was a surge in public care and protection for healthcare workers, creating an integrated, systematic, and efficient social support system^8^. During the formative phase, the social support perceived by healthcare workers may have increased. For example, the doctor-patient relationship improves, and doctor-patient trust increases compared to the pre-epidemic period^9^. Additionally, studies have demonstrated that social support negatively predicts anxiety^10–12^. Social support allows individuals to believe that they are cared for and accepted, reducing individual psychological stress and improving individual psychological well-being^13^. When individuals have close and stable social relationships, they are more likely to feel cared for, giving them more positive resources to cope positively with external stressors and alleviate negative feelings; conversely, individuals who perceive less social support are more likely to interpret their surroundings negatively and respond negatively when encountering negative events^11,12,14^. T The buffering model proposes that social support only works when individuals feel stressed^15^. Based on this, PSS may act as a protective factor for anxiety symptoms during regular epidemic prevention and control. Thus hypothesis 1 is proposed: PSS of health care workers negatively predicts their anxiety symptoms during normalized epidemic prevention and control.

Regarding the mechanisms underlying the onset of emotional stress reactions, a large number of studies have addressed the important role of risk perception on anxiety reactions, and some scholars have even suggested that risk perception plays an important role in each stage of anxiety response generation, presentation, and treatment^16,17^. An individual's perception of external objective risks can be referred to as risk perception, which emphasizes the influence of experiences based on intuitive judgments and subjective feelings on an individual's cognition^18^. Among them, during a pandemic, individuals' intuitive feelings about their infection reflect the emotional characteristics of risk perception (ECRP), which are reflected in a range of emotions such as worry, fear, and even dread^19^. This indicates these emotional characteristics of risk perception negatively to current mental health^20^. While richer social support gives individuals positive emotions and good expectations of things, thereby increasing individuals' adaptability to their environment^7^. As the buffering model proposes that social support regulates individuals' physical and mental health indirectly^15^. Hypothesis 2 was formulated: ECRP mediates health care workers' PSS and anxiety symptoms during regular epidemic prevention and control.

In addition, attention needs to be paid to inter-individual differences when discussing the role of PSS and ECRP in health care professionals. Resilience is a relatively stable personality trait that refers to the developmental phenomenon of individuals who have experienced or are experiencing severe stress/adversity and can demonstrate better adaptation or resilience^21,22^. Resilience is a common adaptive phenomenon^23^ and is essentially a high degree of cognitive flexibility, i.e. the mental ability to adapt thinking to current situations and new and changing contexts^24^. When people are faced with a dilemma, they need to activate relevant cognitive processes and mobilize cognitive resources to facilitate problem-solving, and at the same time flexibly inhibit certain cognitive processes to avoid unnecessary interference^25^. The Hierarchy of Mental Resilience model suggests that individuals with high resilience cope with problems by using positive coping styles and flexibly calling on social support resources^26^. This implies that highly resilient individuals may use social support resources more efficiently to some extent and thus mitigate the emotional characteristics of risk perception more effectively. Therefore, Hypothesis 3 is proposed: Resilience moderates the relationship between PSS and ECRP.

**Figure 1:**
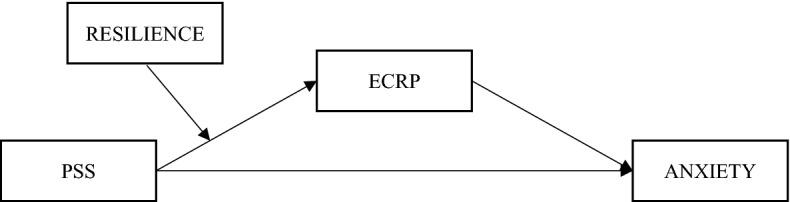
Diagram of the theoretical model.

In summary, based on individual social environmental support systems and intrinsic traits, the study proposes to construct a moderated mediation model (shown in Fig. 1) to explore the relationship between PSS, ECRP, anxiety, and resilience of healthcare workers during the post-epidemic normalization of the COVID-19 outbreak. Specifically, in this model, the effect of PSS on healthcare workers' anxiety is mediated by ECRP, while the mediating pathway (first half of the pathway) is moderated by resilience. This model contributes to understanding the mechanisms underlying the effect of PSS on anxiety in healthcare workers during the normalization of epidemic prevention and control.

## Results

### Test for common method bias

Since the data are cross-sectional, it is possible to use the conduct of the Harman one-factor test to assess common method biases^27^. The results of the Harman one-factor test revealed that a total of 6 factors were generated. The explanation rate of the first factor was 48.374%, which was less than the critical standard of 50%^28^, indicating that there was no obvious common method deviation in this study. These results indicate that the common method is not a problem in this study.

### Descriptive and correlation analyses

The means, standard deviations, and correlation coefficients for PSS, resilience, ECRP, anxiety symptoms, gender, and age are shown in Table 1. PSS, ECRP, resilience, and anxiety symptoms all showed a two-way correlation. To avoid the effect of gender and age, the two variables were treated as control variables in this study. Age was significantly associated with PSS, resilience, and anxiety symptoms, and gender was significantly associated with PSS, resilience, and anxiety symptoms.

**Table 1:** Results of correlation analysis.

Variables	1	2	3	4	5	6
Age	1					
Gender	− 0.218***	1				
PSS	0.086*	− 0.087*	1			
ECRP	0.062	0.000	− 0.164***	1		
Resilience	0.134***	− 0.145***	0.614***	− 0.174***	1	
Anxiety Symptoms	− 0.146***	0.113**	− 0.472***	0.164***	− 0.466***	1
M	37.62		69.39	7.58	102.46	9.58
SD	10.32		13.56	3.17	18.17	3.56

Gender, 1 = male, 0 = female, ^*^P < 0.05, ^***^P < 0.01, ^***^P < 0.001.

### Tests for moderated effects with mediation

First, we conducted a path analysis with PSS as the predictor variable, anxiety as the outcome variable, and ECRP as the mediating variable. Results are shown in Table 2, where, controlling for gender and age, PSS significantly negatively predicted ECRP (b = − 0.18, t = − 5.14; 95% CI = [− 0.25, − 0.11]), adding mediating variables to ECRP significantly positively predicted anxiety symptoms (b = 0.09, t = 2.95; 95% CI = [0.03 , 0.15]) and PSS significantly and negatively predicted anxiety symptoms (b = − 0.45, t = − 14.54; 95% CI = [− 0.51, − 0.39]), suggesting that ECRP partially mediates the relationship between PSS and anxiety, testing Hypotheses 1 and 2.

**Table 2:** Results of the mediating effects model with moderation.

Predictor variables	Outcome variable: ECRP				Outcome variable: anxiety symptoms			
	*b*	*SE*	*P*	95%CI	*b*	*SE*	*P*	95%CI
Constants	− 0.140	0.214	0.515	[− 0.560, 0.281]	0.188	0.186	0.314	[− 0.178, 0.553]
Age	0.008	0.003	< 0.05	[0.001, 0.014]	− 0.010	0.003	< 0.01	[− 0.016, − 0.004]
Gender	− 0.056	0.080	0.485	[− 0.149, − 0.012]	0.109	0.071	0.124	[0.030, 0.247]
PSS	− 0.129	0.045	< 0.01	[− 0.218, − 0.041]	− 0.452	0.031	< 0.001	[− 0.513, − 0.391]
Resilience	− 0.129	0.044	< 0.01	[− 0.215, − 0.043]				
Product term	− 0.081	0.035	< 0.05	[− 0.149, − 0.012]				
ECRP					0.091	0.031	< 0.01	[0.031, 0.152]
Joint explanatory power	*R*^2^ = 0.051***				*R*^2^ = 0.250***			
Model significance	*F*_(5,818)_ = 8.856				*F*_(4,819)_ = 68.276			

***P < 0.001, The product term is the product for PSS and resilience.

**Figure 2:**
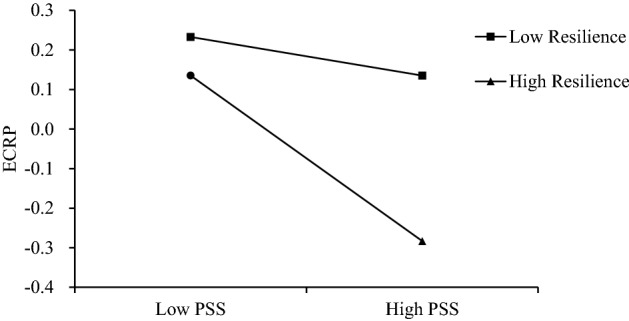
Diagram of the moderating effect.

Second, resilience was included as a moderating variable in the mediation model. The results are shown in Table 2. The interaction term between PSS and resilience was a significant predictor of ECRP (b = − 0.08, t = − 2.31; 95% CI = [− 0.15, − 0.01]), and thus the moderating effect of resilience was significant. To more clearly explain the substance of the interaction effect between resilience and social support, resilience was grouped high and low by the next one standard deviation, a simple slope test was conducted and a simple effect analysis plot was drawn (Fig. 2). The results showed that individuals with high resilience had significantly lower emotional perceptions of epidemic risk with increasing levels of PSS (b = − 0.21, t = − 3.25; 95%CI = [− 0.34, − 0.08]); whereas individuals with low resilience had non-significant changes in ECRP with increasing levels of PSS (b = − 0.05, t = − 1.01; 95% CI = [− 0.14, − 0.04]). Taken together, the moderated model with mediation constructed in this study held (Index =− 0.0073, SE = 0.0044, 95% CI = [− 0.0177, − 0.0003]), and resilience moderated the first half of the mediated pathway of the effect, testing Hypothesis 3.

## Discussion

Using data from Chinese medical personnel one year after the COVID-19 outbreak, this study aimed to determine correlates of PSS, ECRP, resilience, and anxiety symptoms and explore how to alleviate the anxiety symptoms of medical workers from the perspectives of social environment support system and personal traits. Results showed that PSS negatively predicts anxiety symptoms and the prediction mechanism is that the PSS predicts anxiety through the mediating role of ECRP. It is worth noting that resilience modulates the whole model by modulating the first half path of the mediator model. A possible explanation for these results will be discussed below.

The present study found that healthcare workers' PSS had a significant negative effect on anxiety symptoms, which is similar to previous findings^10,11^. For example, Labrague and De Los Santos indicate that social support can alleviate nurses' anxiety about COVID-19^10^. This suggests that PSS alleviates anxiety symptoms in healthcare workers. A well-developed social support system that helps individuals stay healthy or recover from injury^29^. Previous studies have shown that individuals with high PSS can provide themselves with positive psychological resources and reduce stress levels through the sense of satisfaction, belonging and self-worth gained from social interactions^30^. Individuals with low PSS, on the other hand, tend to think in negative terms and are unable to effectively feel many socially supportive events, and are unable to make good use of these positive resources, creating excessive emotional experiences of stress that is detrimental to mental health^11,31^. Therefore, PSS provides a protective effect on anxiety symptoms in healthcare workers during standing epidemic prevention and control where the potential for localized outbreaks of the epidemic exists.

Moreover, this study found that PSS directly influenced anxiety symptoms in healthcare workers and indirectly through ECRP, which explained the mechanism of PSS action anxiety. Blix, Birkeland, and Thoresen found that a higher level of COVID-related worry was significantly associated with a higher level of psychological distress, and a lower level of life satisfaction^20^. Excessive risk perception tends to lead individuals to develop symptoms of depression and anxiety^32^. Moreover, attention to and interpretation of threatening stimuli can cause individuals to develop negative emotions such as worry about reactions that may lead to negative outcomes, and the constant repetition of this process can lead to the development of anxiety symptoms^33^. Current research finds that the PSS can ease this process. The buffering effect mechanism of social support suggests that PSS protects mental health by reducing the negative impact of stressful events on individuals and decreasing the likelihood that stressful events will trigger anxiety and depression^10,31^. So when the emotional feelings of risk assessment decline, the individual's corresponding anxiety symptoms are alleviated.

Additionally, the study considered the role of personal traits and confirmed that resilience is a moderating variable in the relationship between PSS and ECRP, with the moderating effect occurring in the first half of the mediated pathway. Specifically, for individuals with high resilience, emotional perceptions of the epidemic risk decreased significantly with increasing levels of PSS. While for individuals with low resilience, the emotional perceptions did not change significantly with increasing levels of PSS. This suggests that resilience is an important psychological resource in individuals' assessment of risk events, and to some extent increases the protective effect of PSS^34^. The hierarchical model of resilience can explain this result very well. As postulated by the model, when faced with a stressful event, individuals use the psychological resources from the social support of others, family, and community, combined with their psychological resources after being able to adapt well in the face of previous life adversity, trauma, or other major life stressors, to perceive and assess the risky crisis event, allowing negative emotional feelings to decline^2,21^. However, low resilience does not provide sufficient resilience resources to achieve the positive effects of adjunctive social support in reducing ECRP.

In conclusion, the above results provide some positive inspirations for public management. First of all, we should pay attention to the social environment support system of health management workers and realize the positive role of social support. Although the current support system of health care workers has increased, we still need to give them adequate social support such as interpersonal support and organizational support promptly (COVID-19 anxiety among front-line nurses: Predictive role of organizational support, personal resilience, and social support). In addition, we cannot ignore the intrinsic strengths of the individual, such as mental resilience. We found that individuals with high resilience may be somewhat more effective in using social support resources. Resilience is not static and can be enhanced by several interventions^35^. The results of this study support the cognitive-behavioral theory that risk perceptions influence anxiety. Therefore, in the context of the current epidemic situation, we can use digital cognitive behavioral therapy conducted online to alleviate the anxiety symptoms of healthcare workers^36^.

However, there are certain shortcomings in this study. It is a cross-sectional study and has certain limitations in clearly comprehending the causal relationship between PSS and anxiety symptoms among healthcare workers. Follow-up studies could be conducted using a longitudinal design to provide more favorable evidence to reveal the causal relationship between the two. Secondly, our focus on the emotional characteristics of risk perception is more general, and the concept can be refined in the future, such as concern and fear of COVID-19.

## Methods

### Participants

One year after the outbreak of COVID-19, from February 4 to March 1, 2021, 1,430 people were called to participate in the questionnaire survey in Chengdu, Mianyang, and other places in China. Participants must be medical staff and were all involved in the work at the time of the COVID-19 outbreak. There is no extensive epidemic in Sichuan, but there have been large-scale local outbreaks in rural areas of other provinces in China, such as Hebei, Jilin, and Heilongjiang. People perceive the risk of the epidemic, but life has returned to normal. First, this study screened invalid data by the following steps: Remove data with an answer time of fewer than 300 s, find similar (according to IP, submission time, age, residence, etc.) to filter duplicate questionnaires, and the Mahalanobis distance is used to exclude data outside the 0.001 standards. There was 839 medical staff were obtained. Among them, the age of medical staff was 37.62 ± 10.324, with 223 males (26.6%), and 616 females. People (73.4%).

The study was approved by the ethical review committee of the Southwestern University of Science and Technology because it used anonymous and non-harmful social survey methods on the subjects. Otherwise, all methods used in the study were conducted by the relevant guidelines and regulations. All subjects participated voluntarily and signed an informed consent form.

### Research tools

Perceived Social Support Scale (PSS). The PSS Scale^37^ is a 12-item scale, including three dimensions: family support (e.g. "I can talk to my relatives about my issues"), friend support (e.g. "My friends can help me"), and other support (e.g. "Someone is there when I need them"). A 7-point scale is used, with higher scores indicating higher perceptions of social support. In this study, Cronbach's α coefficient for the total scale was 0.932.

Emotional Characteristics of Risk Perception (ECRP). The emotional characteristics of the COVID-19 Risk Perception Scale developed by Xi Juzhe et al.^19^ was used, with 3 questions: "How likely do I think I am to contract Coronavirus?", "I am worried about contracting Coronavirus. And, "I think I am vulnerable to contracting Coronavirus". The options are scored on a 5–6 point Likert scale, with higher scores indicating higher emotional feelings about risk. The scale has good internal consistency reliability, structural validity, and validity scale, and can be used for the scientific assessment of people's risk perception of major public health emergencies^38^. In this study, Cronbach's α coefficient for this scale was 0.822.

Connor-Davidson Resilience Scale (CD-RISC). The Resilience Scale developed by Connor et al. was used to assess positive psychological qualities that facilitate adaptation to adversity^39^, with 25 items (e.g. "I can handle whatever happens in my life") on a 5-point scale. The higher the score, the higher the level of resilience. In this study, Cronbach's α coefficient for this scale was 0.971.

Generalized Anxiety Disorder 7 (GAD-7). The Anxiety Symptom Scale, recommended by the Diagnostic and Statistical Manual of Mental Disorders, 5th edition, was used as a valid instrument to measure anxiety symptoms^40^. It is used to assess the frequency of anxiety symptoms over the past 2 weeks, consisting of 7 questionnaire items (e.g. "Feeling afraid that something terrible is going to happen") on a 4-point scale, with higher scores indicating more intense anxiety symptoms. In this study, Cronbach's α coefficient for this scale was 0.944.

### Data processing

All data were analyzed using IBM SPSS Statistics 23.0. We used an independent sample T-test to investigate the difference in perceived social support between medical staff and civil servants. Then, the raw data of the perceived social support Scale, the emotional sensation of risk Scale, the Connor-Davidson resilience scale, and generalized anxiety disorder 7 were standardized. And the raw data for PSS, resilience, ECRP, and anxiety symptoms were standardized. Then we used Model 4 of the Hayes PROCESS macro^41^ to examine the mediation effect. Moreover, Model 7 of the PROCESS macro was used to test whether resilience moderated the mediation process. Bootstrapping (5000 bootstrap samples) with 95% confidence intervals (CIs) was conducted to test the significance of indirect effects^41^. The 95% CIs and Index did not include zero, indicating a significant effect^42^.

## Data Availability

The data supporting the finding of this study are available on request from the corresponding author.
